# Enteric Polymer–Based Amorphous Solid Dispersions Enhance Oral Absorption of the Weakly Basic Drug Nintedanib via Stabilization of Supersaturation

**DOI:** 10.3390/pharmaceutics14091830

**Published:** 2022-08-30

**Authors:** Yuling Qin, Chuyao Xiao, Xiaoyue Li, Jiangeng Huang, Luqin Si, Minghui Sun

**Affiliations:** 1 Department of Pharmacy, Tongji Hospital, Tongji Medical College, Huazhong University of Science and Technology, Wuhan 430030, China; 2 School of Pharmacy, Tongji Medical College, Huazhong University of Science and Technology, Wuhan 430030, China

**Keywords:** nintedanib, amorphous solid dispersion, supersaturation, enteric polymer, pharmacokinetics

## Abstract

The pH–induced crystallization of weakly basic drugs in the small intestine limits oral bioavailability. In this study, we investigated the solubilization and inhibitory effects on nintedanib in the presence of enteric polymers (HPMCAS LG, HPMCAS MG, Eudragit L100 55, and Eudragit L100). These polymers provided maintenance of supersaturation by increasing the solubility of nintedanib in PBS 6.8 in a concentration-dependent manner, and the improved ranking was as follows: Eudragit L100 > Eudragit L100 55 > HPMCAS MG > HPMCAS LG. After being formulated into amorphous solid dispersions (ASDs) by a solvent evaporation method, the drug exhibited an amorphous state. The pH shift dissolution results of polymer-ASDs demonstrated that four polymers could effectively maintain the drug supersaturation even at the lowest ratio of nintedanib and polymer (1:1, *w*/*w*). Eudragit L100–ASD could provide both acid resistance and the favorable mitigation of crystallization in GIF. In comparison to the coarse drug, the relative bioavailability of Eudragit L100–ASD was 245% after oral administration in rats, and *T*_max_ was markedly delayed from 2.8 ± 0.4 h to 5.3 ± 2.7 h. Our findings indicate that enteric ASDs are an effective strategy to increase the intestinal absorption of nintedanib by improving physiologically generated supersaturation and subsequent crystallization.

## 1. Introduction

Nintedanib, a tyrosine kinase inhibitor, was firstly approved by the FDA in 2014 for treatment in idiopathic pulmonary fibrosis (IPF) [1]. Besides the role in fibrosis, nintedanib possesses broad-spectrum inhibitory activities on the angiokinase of growth factor receptors, including VEGFR1–3, PDGFRα/β, and FGFR1–4, as well as of RET, FLT-3, and Src non-receptor tyrosine kinases [2]. The multi-targeting profile provides nintedanib with the potential to effectively prevent tumor growth and metastasis. Moreover, the high specificity of nintedanib can minimize drug toxicity and resistance development across a broad range of cancers, where other anti-angiogenic agents have failed [3,4]. Clinically, the weakly basic drug nintedanib is orally administered in soft capsules (Ofev^®^ and Vargatef^®^) for twice-daily dosing. Although nintedanib is easily soluble in the stomach, it still shows low oral bioavailability (around 4.7%) due to poorly aqueous solubility (circa 11.98 µg/mL) under pH 6.8 [5], efflux by P-gp [6], and first-pass metabolism [7]. Recently, several novel formulations, such as self-microemulsifying drug delivery systems [5], sustained-release nano-delivery systems [8], and nanocrystals [9], have been reported, with an increase in oral bioavailability from 1.6- to 2.5-fold compared to that of oral solutions or capsules.

Nintedanib, also known as BIBF1120, has the chemical name 1H-indole-6-carboxylic acid, 2,3-dihydro-3-[[[4-[methyl[(4-methyl-1-piperazinyl)acetyl]-amino] phenyl]amino]phenylmethylene]-2-oxo-,methyl ester, (3Z)-, ethanesulfonate [1]. It displays a pH-dependent solubility profile. The solubility of nintedanib at pH values below 4.5 is nearly 5.0 mg/mL but it is insoluble above 6.0 [5]. Although nintedanib salt easily dissolves in acidic fluid, precipitation or recrystallization is prone to occur when the pH increases in the intestine tract. As the small intestine is the main site of drug absorption, maintenance of a dissolved state in the intestine is more critical than in the stomach. Consequently, appropriate precipitation inhibitors are necessary to slow the decline of the supersaturation state via interfering with nucleation and/or crystal growth.

Amorphous solid dispersions (ASDs) are one of the most frequently used methods for improving the oral bioavailability of poorly soluble drugs [10]. Hydrophilic polymers such as polyvinyl pyrrolidone (PVP), polyethyleneglycols (PEG), and Soluplus^®^ (SOL) are commonly chosen for their better wettability and dispersibility to obtain immediate release characteristics in ASDs [11,12,13,14]. These carriers could promote temporary supersaturation and provide the driving force to enhance drug absorption [15]. However, a drug solution in the supersaturated state possesses higher chemical potential and tends to precipitate into an energetically more favorable crystalline form [16,17,18]. Moreover, a faster rate of supersaturation generation causes a sharper drop in drug concentration due to rapid precipitation [19]. It has been reported that the rate of dissolution in simulated gastric fluid was inversely proportional to in vivo drug absorption [20,21]. To benefit from the supersaturated state, temporary inhibition of precipitation is necessary to maintain the elevated concentrations for a sufficient period of absorption time. Interestingly, polymers with pH-dependent solubility lead to higher oral bioavailability as compared with ASDs based on immediate-release polymers [22], which may be attributed to the balance ability of these polymers between the rate of supersaturation generation and the precipitation kinetics. Han et al. showed that the true solubility advantage of ASDs depended on the critical supersaturation, below which precipitation is not observed for a sufficiently long period [13].

In this study, we investigated the effects of pH-dependent soluble polymers, namely hydroxypropylmethylcellulose acetate succinate (HPMCAS LG and MG) and methacrylic acid ethyl acrylate copolymer (Eudragit L100 55 and L100), on the solubility of nintedanib and supersaturation maintenance. ASDs were formulated with four different polymers and characterized by differential scanning calorimetry (DSC), powder X-ray diffraction (PXRD), scanning electron microscopy (SEM), and Fourier-transform infrared spectroscopy (FT–IR). After the in vitro dissolution behaviors were compared in a pH shift non-sink condition, the in vivo pharmacokinetics of nintedanib and Eudragit L100–ASD with the preferred enteric-release profile were examined to evaluate the enhanced absorption. In addition, the physical stability of the prepared solid dispersions was investigated under high temperatures and high humidity.

## 2. Materials and Methods

### 2.1. Materials

**Figure 1:**
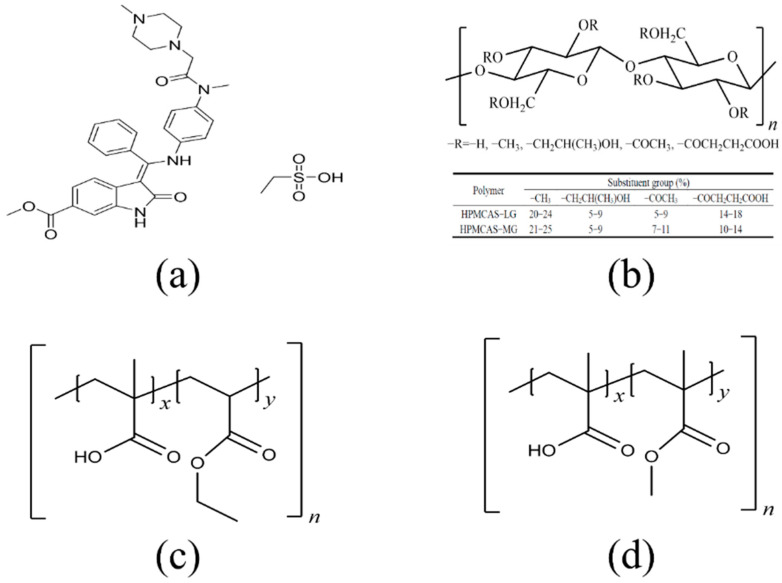
Molecular structures of (**a**) nintedanib (pKa = 7.9), (**b**) HPMCAS (LG and MG), (**c**) Eudragit L100 55, (**d**) Eudragit L100.

Nintedanib was purchased from Nanjing Core Tech Biomedical Co., Ltd. (Nanjing, China). HPMCAS (grade LG and MG) was kindly provided by Shin Etsu (Shin Etsu Chemical Co., Ltd., Tokyo, Japan). Eudragit^®^ L100 55 and Eudragit^®^ L100 were donated by Evonik Industries (Darmstadt, Germany). The chemical structures of nintedanib and polymers are summarized in Figure 1. All other reagents were of analytical grade and used as received.

### 2.2. Determination of Equilibrium Solubility and Apparent n–Octanol/Water Partition Coefficient of Nintedanib

An excess amount of nintedanib powder was added to simulated gastric fluid (pH 1.2) and PBS buffer solutions at pH 4.5, 5.5, 6.0, 6.8, and 7.4. The suspension was continuously shaken and balanced at 37 °C for 72 h. After centrifugation at 13,000 rpm for 10 min, the supernatant was diluted with mobile phase for quantitative determination by HPLC using a Shimadzu LC-20AT chromatographer (Shimadzu Corporation, Japan) equipped with a UV detector set at 382 nm. The stationary phase comprised a C18 analytical column (Aligent Eclipse XDB-C18, 4.6 × 250 mm, 5 µm), maintained at 40 °C. The mobile phase consisted of methanol and 0.1% phosphate in water (65:35, *v*/*v*) at a flow rate of 1 mL/min. The injection volume was 20 µL.

The apparent n–octanol/water partition coefficients (*D*) of nintedanib were determined by the shake flask method. Nintedanib was precisely weighed and dissolved in different medium-saturated n–octanol to obtain the nintedanib/n–octanol solution. The resulting solution was vortexed with an equivalent volume of n–octanol–saturated aqueous media for 5 min. The mixture was further shaken at 37 °C for 24 h to reach equilibrium. After separating the water phase and oil phase by centrifugation for 15 min at 3500 rpm, the drug concentrations were assayed by HPLC. The values of log *D* were calculated as follows.

Log *D* = *C*_o_/*C*_w_

where *C*_o_ represents the concentration of nintedanib in the n–octanol phase, and *C*_w_ represents the concentration of nintedanib in the aqueous phase.

### 2.3. Solubility Measurement of Nintedanib in Polymer Solution

The equilibrium solubility of nintedanib in different polymer solutions was also determined using the shake flask method [23]. Briefly, an excess amount of nintedanib powder was dispersed in 5 mL pH 6.8 PBS buffer without or with pre-dissolved polymers (0.1%, 0.5%, or 1% *w*/*v*). Then, the suspension was shaken in a water bath under 37 °C at 100 rpm for 72 h. The mixture was centrifugated at 37 °C and 13,000 rpm for 10 min. The supernatant was filtrated through a 0.22 µm polyvinylidene fluoride filter. The filtrates were diluted with mobile phase and quantified by the HPLC method, as described above.

### 2.4. In Vitro Supersaturation Studies

Supersaturation studies were performed in PBS 6.8 medium containing 0, 0.1%, 0.5%, and 1.0% of HPMCAS LG, MG, Eudragit^®^ L100 55, or Eudragit^®^ L100 [24,25]. Nintedanib was dissolved in DMSO at a concentration of 80 mg/mL. Then, 50 µL of nintedanib solution was added to 5 mL of polymer solution and agitated at 100 rpm under a 37 °C water bath. Then, 300 µL aliquots were taken at 5, 15, 30, 60, 90, 120, 180, 240, 300, and 360 min and centrifugated at 13,000 rpm for 10 min, respectively. The supernatants were diluted in methanol and quantified by the HPLC method. The values of AUC_0–360min_ were calculated using GraphPad Prism 8 software (GraphPad Inc., San Diego, CA, USA) to assess the maintenance effects of polymers on the extent of supersaturation in the solutions. A one-way analysis of variance (ANOVA) followed by Tukey’s multiple comparisons was employed to test the statistical significance of the AUC values.

### 2.5. Polarized Light Microscopy

Polarized light microscopy (PLM) was applied to observe the crystallization behavior of nintedanib in supersaturation studies [25,26]. Aliquots were withdrawn and centrifuged (5000 rpm, 5 min) at 5, 60, 120, 240, and 360 min. Images of the crystallization of nintedanib in the supernatant were recorded using a polarization microscope with a 40× magnification objective (CKX53 microscope with polarizer, Olympus Corporation, Tokyo, Japan).

### 2.6. Preparation and Characterization of Amorphous Solid Dispersions

#### 2.6.1. Preparation of Amorphous Solid Dispersions

ASDs of nintedanib with HPMCAS LG, HPMCAS MG, Eudragit^®^ L100 55, or Eudragit^®^ L100 were prepared using a solvent evaporation method, as described previously, with a slight modification [27]. Briefly, nintedanib and different polymers in different ratios (1:1, 1:3, or 1:5, *w*/*w*, drug: polymer) were dissolved in methanol by stirring. The organic solvents were removed by rotary evaporation at 60 °C. The obtained films were dried overnight under a vacuum at room temperature. After being ground with a mortar and pestle, the ASDs were forcibly passed through a 200-mesh sieve (75 µm). The resulting ASD powders were stored in a desiccator at ambient temperature until analysis.

#### 2.6.2. Scanning Electron Microscopy (SEM)

The morphologies of the coarse drug, polymers, physical mixtures, and ASDs were observed using a field-emission scanning electron microscope (Nova Nano SEM 450, FEI, Eindhoven, The Netherlands) at a 10.0 kV acceleration voltage. Small quantities of powder samples were mounted and coated with a thin gold-palladium layer. Photographs were recorded to characterize the surface shapes of powders.

#### 2.6.3. Differential Scanning Calorimetry (DSC)

A thermodynamic test was performed using a DSC instrument (TA, New Castle, DL, USA). After weighing approximately 5 mg powdered samples into aluminum pans, the samples were scanned in a nitrogen atmosphere at a heating rate of 10 °C/min from 30 °C to 320 °C. The existing state of nintedanib was analyzed from the endothermic peak of the recorded DSC curve.

#### 2.6.4. Powder X-ray Diffraction (PXRD)

The diffraction patterns of the coarse drug, polymers, physical mixtures, and ASDs were analyzed using an X-ray diffractometer (Panalytical, Holland) with Cu-Kα radiation at 30 mA and 30 kV. Samples were scanned in the 2*θ* range from 5° to 45° at a rate of 5°/min.

#### 2.6.5. Fourier Transform Infrared Spectroscopy (FT–IR)

FT–IR (Bruker Corporation, Switzerland) was employed to investigate the intermolecular interactions of nintedanib and polymers. The solid dispersions and corresponding polymers were mixed with KBr and then tableted. FT–IR spectra were collected over the wave number range of 500–4000 cm^−1^ with a resolution of 4 cm^−1^.

### 2.7. pH Shift Dissolution Studies of Amorphous Solid Dispersions

pH shift dissolution was conducted by the paddle method with a rotation speed of 50 rpm using a dissolution tester (ZRS-4, Tianjin University Radio Factory, China) in simulated gastric fluid (SGF, pH 1.2) and simulated intestinal fluid (SIF, pH 6.8). The ASDs were initially placed in 750 mL of SGF at 37 °C ± 0.5 °C and stirred for 120 min. Then, pH was switched rapidly to 6.8 by adding 250 mL 0.2 mol/L sodium phosphate solution. The samples were agitated for another 240 min. Aliquots of 2 mL were withdrawn at predetermined time intervals (5, 15, 30, 60, 90, 120, 150, 180, 240, 300, and 360 min) and replenished with equal volumes of fresh media. The samples were immediately filtered through a 0.22 µm membrane and diluted in the mobile phase for HPLC quantification of nintedanib.

### 2.8. Stability Study

The physical stability of the ASDs (1:5, *w*/*w*, drug: polymer) was investigated under high humidity (RH 92.5 ± 5%, 25 ± 1 °C) and high temperature (60 ± 1 °C, RH 50 ± 5%), respectively. Samples were collected at 5 and 10 days and characterized by PXRD. The dissolution profiles were compared with the performance of the ASDs at time 0 using the similarity factor *(f*_2_) [28]:

$$f_{2}=50\times \log\left\{{\left[1+\left(1/n\right)\sum_{t=1}^{n}(R_{t}-T_{t}){}^{2}\right]}^{-0.5}\times 100\right\}$$

where *n* is the number of time points; *R_t_* and *T_t_* correspondingly represent the dissolution value of the reference and test at time *t*. The release profiles were similar if *f*_2_ > 50. No more than one measurement should be considered after 85% dissolution of the two contrastive formulations.

### 2.9. In Vivo Pharmacokinetic Study

**Animals.** Rats are commonly used for preclinical in vivo pharmacokinetic studies. Male Sprague-Dawley (SD) rats weighing 180–220 g were supplied by the laboratory animal center of Tongji Medical College, Huazhong University of Science and Technology (Wuhan, China). The rats were maintained in a room (20–25 °C and 50–60% humidity) under a 12 h light/dark cycle, with free access to standard rodent chow and clean water for 7 d before the commencement of the experiment.

**Pharmacokinetic study** [29]. The SD rats were randomly divided into two groups (*n* = 6) and fasted for 12 h before oral administration. The Eudragit^®^ L100 solid dispersion (1:5, *w*/*w*) and the coarse drug (75 μm, 50 mg/kg) were freshly suspended in 0.5% tragacanth gum (*w*/*v*) at a dose volume of 10 mL/kg. Blood samples (0.15 mL blood per time point) were collected into heparinized tubes from the jugular vein pre-dose (0 h) and post-dose (1, 1.5, 2, 2.5, 3, 3.5, 4, 6, 8, 10, 12, 14, and 24 h). The plasma samples were separated by centrifugation at 4000 rpm for 15 min and stored at −80 °C before analysis.

**Plasma sample pretreatment.** All plasma samples were processed by a protein precipitation method. An aliquot of 30 μL plasma was spiked with 150 μL acetonitrile, which contained 250 ng/mL of carbamazepine as an internal standard (IS). The mixture was vortex-mixed for 5 min, followed by centrifugation at 15,000 rpm and 4 °C for 10 min. A volume of 10 μL of supernatant was injected into the ultrahigh-performance liquid chromatography-tandem mass spectrometry (UPLC–MS/MS) system for analysis.

**Quantification of nintedanib**. A prominence UFLC system (Shimadzu Corporation, Kyoto, Japan) with an electrospray ionization (ESI) source in positive mode was used. The separation of analytes was performed on an Ultimate^®^XB–C18 column (2.1 × 50 mm, 5 µm, Welch, Shanghai, China). The column temperature was maintained at 40 °C. The gradient elution was conducted with solvent A (water with 0.1% formic acid) and solvent B (acetonitrile with 0.1% formic acid) at a flow rate of 0.5 mL/min. The gradient program was set as follows: 20% B for 0.10 min; 20% to 65% B from 0.10 to 1.60 min; 65% to 95% B from 1.60 to 1.80 min; 95% B from 1.80 to 3.00 min; 95% to 20% B from 3.00 to 3.20 min; 20% B from 3.20 to 4.00 min. The injection volume was 10 µL, and the autosampler temperature was maintained at 4 °C. The source parameters were optimized: curtain gas: 16 psi, collision gas: medium, ion spray voltage: 5500 V, temperature: 500 °C, nebulizer gas: 50 psi, auxiliary heater gas: 50 psi. Other optimum parameters are listed in Table 1.

**Pharmacokinetic data analysis and statistical analysis** [30]. The calibration curve ranging from 1 to 500 ng/mL was fitted using a weighted (1/x^2^) least-squares linear regression of peak area ratios against concentrations. The plasma concentration-time profiles were analyzed using a non-compartmental model. The main PK parameters, including time to reach *C*_max_ (*T*_max_), maximum plasma concentration (*C*_max_), the area under the plasma concentration-time curve (AUC_0–24h_), half-life (*t*_1/2_), and mean retention time (MRT) were obtained by Phoenix WinNonlin software (Version 6.4, Pharsight Corporation, Mountain View, CA, USA). A two-tailed Student’s *t*-test was adopted to compare the PK parameters of the nintedanib group and Eudragit L100–ASD group. The relative oral bioavailability (*F*_rel_) was calculated by the ratio of AUC_0–24h_ for the ASD and the coarse drug. Differences were considered statistically significant at *p* < 0.05.

## 3. Results and Discussion

### 3.1. Equilibrium Solubility and Apparent n–Octanol/Water Partition Coefficient of Nintedanib

**Figure 2:**
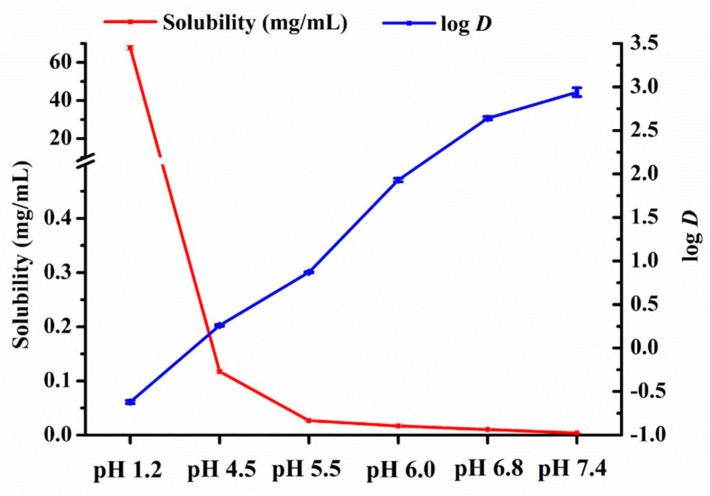
Equilibrium solubility and apparent n–octanol/water partition coefficient for nintedanib in different pH media (mean ± SD, *n* = 3).

The equilibrium solubility and apparent n–octanol/water partition coefficient of a drug in the GI tract are the key parameters that affect drug absorption. As shown in Figure 2, the maximum solubility was 67.7 ± 1.2 mg/mL in pH 1.2 SGF. Sharply decreased solubility of nintedanib was observed when the pH value of the solvent ranged from 1.2 to 5.5. As the pH increased from 5.5 to 7.4, the drug solubility gradually decreased to 26.8 ± 1.6 µg/mL and 3.8 ± 0.2 µg/mL, respectively. The solubility changes with pH value indicated the pH-dependent solubility of nintedanib. On the contrary, the apparent n–octanol/water partition coefficient (log *D* = 2.64 ± 0.02) in the pH 6.8 medium was higher than that (log *D* = −0.62 ± 0.02) in the pH 1.2 medium, indicating that nintedanib was more lipophilic in the neutral environment than in the acidic environment.

### 3.2. Effect of Enteric Polymers on Solubility of Nintedanib

**Figure 3:**
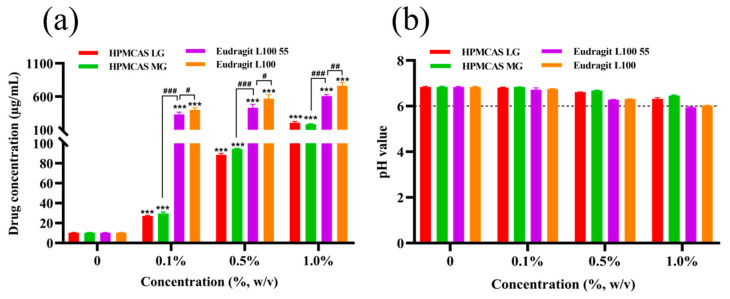
Nintedanib solubility (**a**) and pH values (**b**) of PBS buffer in presence and absence of polymers at different concentrations (0.1%, 0.5%, and 1.0% *w*/*v*) (*n* = 3). *** *p* < 0.001 significantly compared to the blank polymer group. ^#^ *p* < 0.05, ^##^ *p* < 0.01, ^###^ *p* < 0.001.

The effect of different polymers on the solubility of nintedanib is shown in Figure 3a. The addition of enteric polymers in pH 6.8 PBS buffer ranging from 0.1% to 1% (*w*/*v*) significantly increased the apparent solubility of nintedanib in a concentration–dependent manner (*p* < 0.001). Compared to pH 6.8 buffer without polymers, HPMCAS LG, HPMCAS MG, Eudragit L100 55, and Eudragit L100 at a concentration of 0.1% (*w*/*v*) elevated drug solubility 2.6-, 2.9-, 32.5-, and 39.0-fold, respectively. At the concentration of 0.5%, Eudragit L100 increased the drug solubility 6.4-, 6.0-, and 1.3-fold in comparison with HPMCAS LG, HPMCAS MG, and Eudragit L100 55, respectively. HPMCAS LG presented a similar increase as HPMCAS MG (*p* > 0.05). Eudragit L100 and L100 55 had similar potential to solubilize nintedanib, while Eudragit L100 showed stronger solubilization (*p* < 0.05 or *p* < 0.01). Compared to Eudragit L100 and L100 55, the improvement in the drug solubility was more dependent on the concentration gradient of HPMCAS LG and MG (*p* < 0.001). As shown in Figure 3b, the pH value of the corresponding medium slightly decreased with the additional content of polymers. However, the pH values were still around 6.0 at a 1.0% polymer concentration. These data indicated that the interaction between the enteric polymers and the drug improved the solubility of nintedanib, other than changing the pH value of the environment.

### 3.3. Supersaturation Kinetics of Nintedanib in the Presence of Enteric Polymers

**Figure 4:**
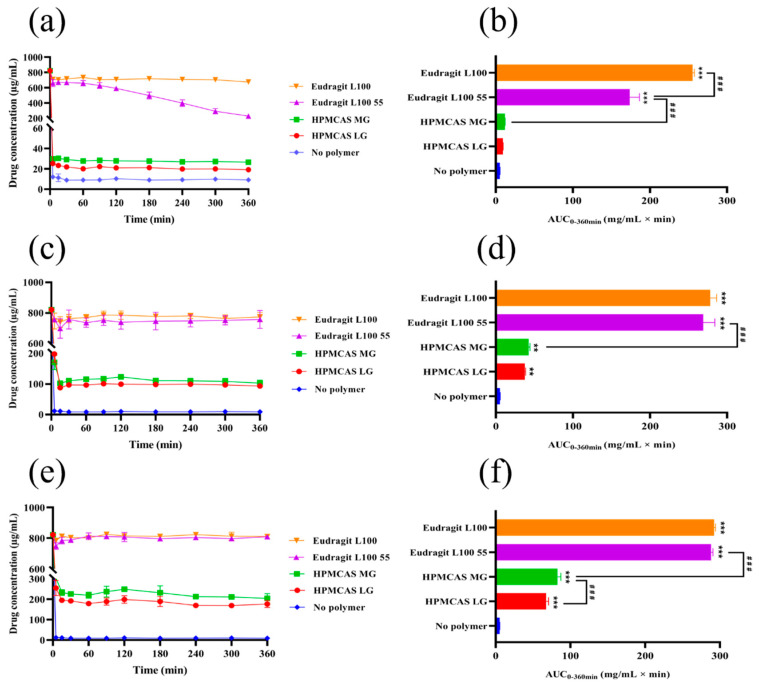
Supersaturation kinetics of nintedanib and AUC_0–360min_ in pH 6.8 PBS buffer with different polymers. (**a**,**b**) 0.1%; (**c**,**d**) 0.5%; (**e**,**f**) 1% (*w*/*v*). Data are expressed as the mean ± SD (*n* = 3). ** *p* < 0.01, *** *p* < 0.001 significantly compared to the no polymer group, ^###^ *p* < 0.001.

The in vitro nintedanib concentration-time profiles and their respective AUC_0–360min_ are shown in Figure 4. Upon inducing the supersaturation of nintedanib without polymers, immediate and complete precipitation could be observed. When enteric polymers were added to pH 6.8 PBS buffer, the remaining concentration of nintedanib increased with the polymer concentration increase. The addition of HPMCAS increased the apparent solubility of nintedanib but to a lower extent (Figure 4a,b). The AUCs in the medium with over 0.5% of HPMCAS LG were statistically different from those for PBS (*p* < 0.01 or *p* < 0.001). Compared to HPMCAS LG, a marked increase in AUC in the HPMCAS MG groups at 1.0% was observed (*p* < 0.001). Moreover, Eudragit L100 55 at 0.1% maintained a drug concentration of approximately 650 μg/mL for up to 60 min but decreased later (Figure 4a). With the increase in the Eudragit L100 55 concentration, the ability to maintain supersaturation increased (Figure 4c,e). Among all four polymers, Eudragit L100 demonstrated the best supersaturation maintenance in PBS 6.8, reaching the highest AUC (Figure 4b,d,f). In particular, Eudragit L100 at 0.1% was able to maintain a drug concentration of circa 670 μg/mL within 360 min and obtained the highest AUC (*p* < 0.001). These findings suggested a certain concentration-dependent effect of the four polymers on maintaining drug supersaturation and showed that the capability of retaining supersaturation is in the order Eudragit L100 > Eudragit L100 55 > HPMCAS MG > HPMCAS LG.

The influences of the four enteric polymers on the supersaturation kinetics of nintedanib were consistent with the impacts on drug solubility. HPMCAS LG and Eudragit L100 55 dissolved at pH ≥ 5.5, while both HPMCAS MG and Eudragit L100 dissolved at pH ≥ 6.0, with pH-dependent solubility. As the pH values of drug-polymer solutions were weakly affected (Figure 3b), the polymer-induced improvement in solubility was not derived from the formation of soluble salt, but rather from a strong interaction between the drug and polymers. Polymer structure, especially the functional groups, has been reported to affect the preservation of the supersaturated state [31]. HPMCAS LG and HPMCAS MG possess both hydrophilic groups and hydrophobic groups. It was found that the ability of HPMCAS to inhibit drug crystallization strongly depended on the succinoyl substituent level in HPMCAS [32]. The increase in the succinoyl substituent ratio of HPMCAS (LG: 0.37, MG: 0.26, average number/glucose ring unit), which strongly affected the hydrophilicity of the polymer due to the ionization of carboxylic acid, reduced the inhibition efficiency for drug precipitation [32]. Adsorption of the polymers’ hydrophobic groups onto the surface of hydrophobic nintedanib could suppress the crystal growth of the drug [33,34]. Similarly, the dependence on the hydrophobicity among HPMCAS and Eudragit might elucidate the inhibitory effects on precipitation. Eudragit L100 55 is a copolymer of methacrylic acid (MA) and ethyl acrylate (EA) (1:1), while Eudragit L100 has the same ratio between MA and methyl methacrylate (MMA) (1:1). Eudragit L100, with more hydrophobic MMA monomers, is more hydrophobic than Eudragit L100 55 [33]. The increased hydrophobicity of Eudragit polymers elevated the inhibition of recrystallization, which is in agreement with a reported study [35].

### 3.4. Crystallization Kinetics of Nintedanib in the Presence of Enteric Polymers

**Figure 5:**
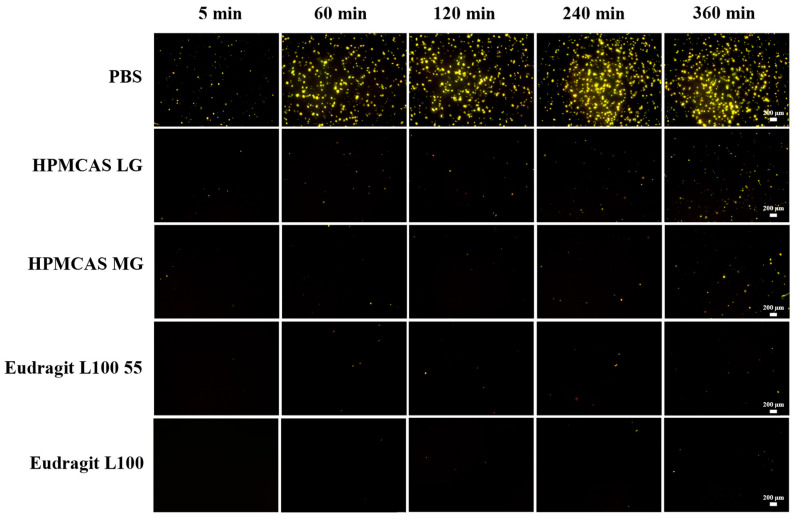
Crystallization of nintedanib in PBS with different polymers (scale bar = 200 μm).

Micro-sized particles formed in the supersaturation solution were detected by polarized light microscopy. As shown in Figure 5, continuous precipitation of nintedanib was observed within 360 min. However, the rate of crystallization differed among different groups. Precipitation of nintedanib particles appeared 5 min after the addition of drug solution in the blank pH 6.8 PBS buffer, and a large number of crystals with a growing size precipitated within 60 min. Although the crystallization of nintedanib still appeared at 5 min, the number of crystals decreased sharply at all specified times in the presence of HPMCAS LG, HPMCAS MG, or Eudragit L100 55. Eudragit L100 could prevent crystallization at 5 min, and the number of nintedanib crystals presented in 360 min was less than that in the other three polymers, indicating the best inhibition of nintedanib precipitation. The trend of maintaining supersaturation was the same as the supersaturation kinetics of the concentration-time profiles.

### 3.5. Characterization of Amorphous Solid Dispersions

#### 3.5.1. SEM

**Figure 6:**
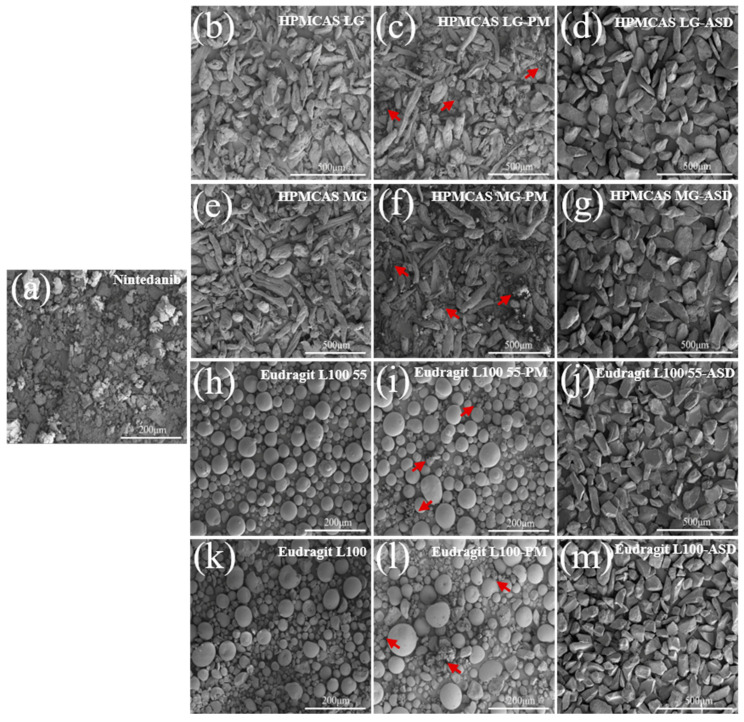
SEM images of (**a**) nintedanib, (**b**) HPMCAS LG, (**c**) HPMCAS LG–PM, (**d**) HPMCAS LG–ASD, (**e**) HPMCAS MG, (**f**) HPMCAS MG–PM, (**g**) HPMCAS MG–ASD, (**h**) Eudragit L100 55, (**i**) Eudragit L100 55–PM, (**j**) Eudragit L100 55–ASD, (**k**) Eudragit L100, (**l**) Eudragit L100–PM, (**m**) Eudragit L100–ASD. Arrows indicate drug crystal structures.

The morphologies of nintedanib, polymers, physical mixtures, and ASDs are illustrated in Figure 6. Nintedanib powder appeared in an irregular shape (Figure 6a). HPMCAS LG and HPMCAS MG exhibited rod-shaped particles with rough surfaces (Figure 6b,e). Eudragit L100 55 and Eudragit L100 showed spherical particles with smooth surfaces (Figure 6h,k). The drug particles dispersed around the polymers for all physical mixtures (PMs, Figure 6c,f,i,l). On the contrary, no drug crystals existed after formulation into ASDs (Figure 6d,g,j,m). Compared to the individual components and physical mixtures, all the ASDs presented uniform texture granules with a drastic change in appearance, suggesting the formation of a new solid phase.

#### 3.5.2. PXRD

**Figure 7:**
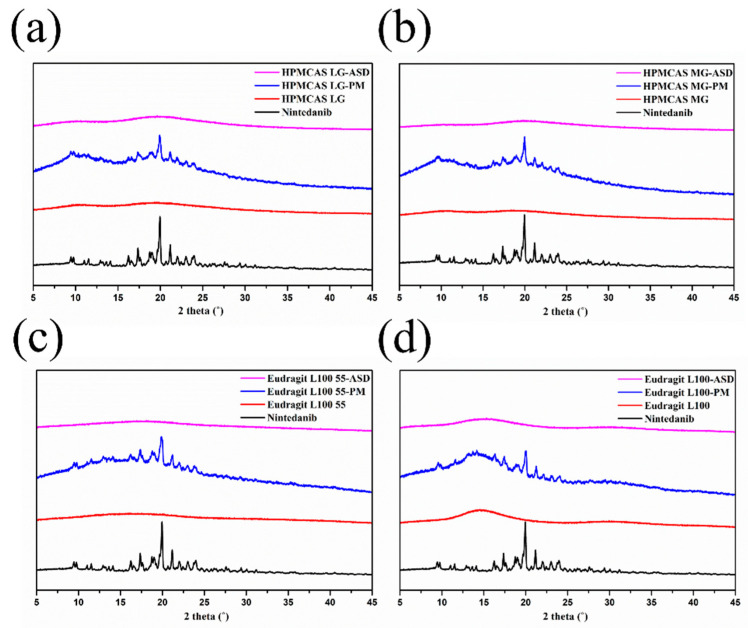
PXRD diffractograms of nintedanib and samples with (**a**) HPMCAS LG, (**b**) HPMCAS MG, (**c**) Eudragit L100 55, (**d**) Eudragit L100.

The PXRD patterns for nintedanib, polymers, physical mixtures, and ASDs in Figure 7 indicate changes in the crystalline structure of the initial substance. The diffractograms of nintedanib presented distinctive peaks at 2θ angles of 11.55°, 17.38°, 18.77°, 19.02°, and 19.95°, which revealed its crystalline state. The PXRD spectra of all polymers exhibited a halo pattern, indicating their amorphous state. For physical mixtures, a series of diffraction peaks superimposed with nintedanib and some diffraction peaks showed a slight reduction due to the dilution of polymers. On the contrary, the sharp diffraction peaks of nintedanib disappeared in all ASDs, demonstrating that nintedanib converted from a crystalline state to an amorphous form.

#### 3.5.3. DSC

**Figure 8:**
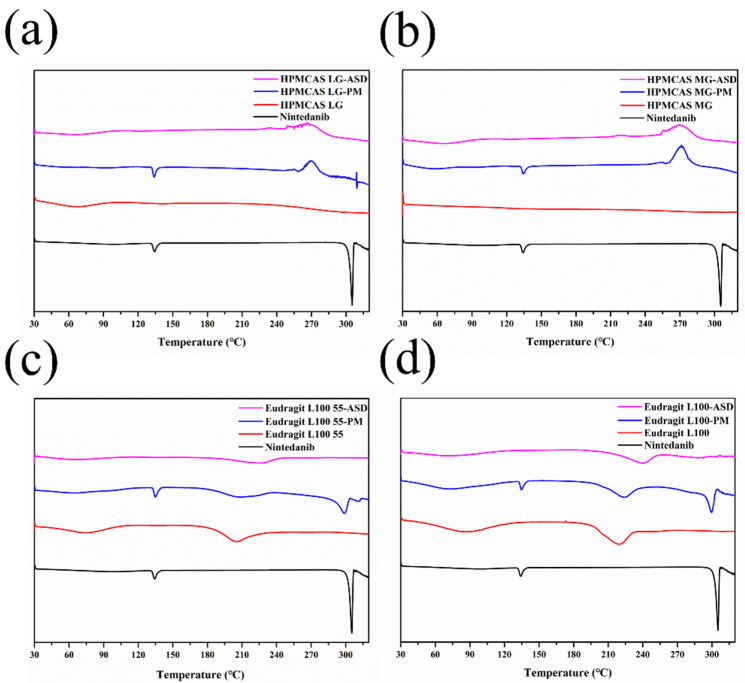
DSC curves of nintedanib and samples with (**a**) HPMCAS LG, (**b**) HPMCAS MG, (**c**) Eudragit L100 55, (**d**) Eudragit L100.

Figure 8 shows the thermal behaviors of nintedanib, polymers, physical mixtures, and ASDs. Nintedanib powder exhibited a small melting peak at 134 °C and a sharp and intense endothermic peak at 305 °C, indicating that the drug existed in a crystalline form, as previously reported [9]. The physical mixtures with HPMCAS LG or HPMCAS MG demonstrated an endothermic peak at 134 °C, whereas the endothermic peak at 305 °C disappeared, which might be attributed to the poor thermal stability of the physical mixtures as HPMCAS degraded above 250 °C [36]. The mixtures of nintedanib with Eudragit L100 55 or Eudragit L100 displayed a shift in endothermic peak to a slightly lower temperature of 300 °C, which might be due to the solvent effect of the molten polymer [37]. No endothermic peaks were detected in all ASDs, suggesting that nintedanib was miscible with the four polymers and converted from the crystalline state to an amorphous state.

#### 3.5.4. FT–IR

**Figure 9:**
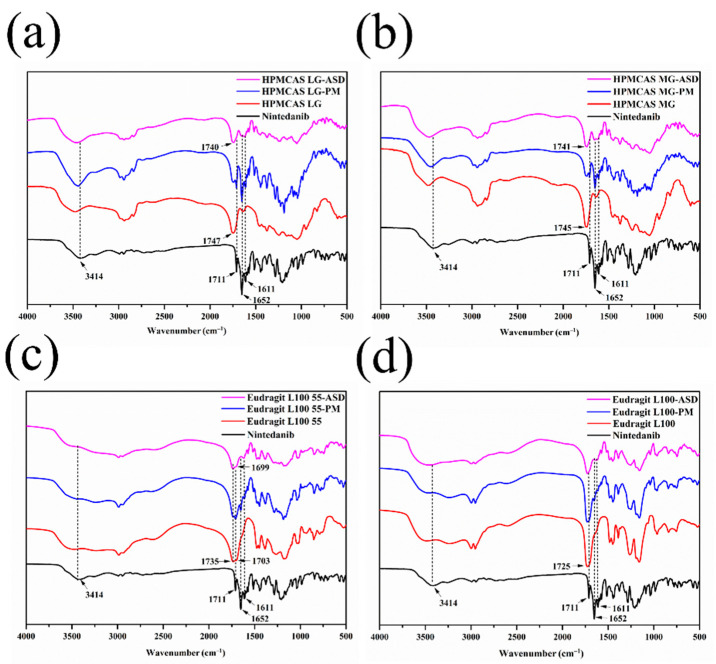
FT–IR spectra of nintedanib and samples with (**a**) HPMCAS LG, (**b**) HPMCAS MG, (**c**) Eudragit L100 55, (**d**) Eudragit L100.

FT–IR analysis was performed for the possible molecular interactions between the drug and polymers. The IR spectra of nintedanib, polymers, PMs, and ASDs are depicted in Figure 9. Characteristic peaks of nintedanib appeared at 3414 cm^−1^ (N–H stretch), 1711 cm^−1^ (C=O stretch, ester), 1652 cm^−1^ (C=O stretch, secondary amide), and 1611 cm^−1^ (C=O stretch, tertiary amide). Physical mixtures of nintedanib with HPMCAS LG, HPMCAS MG, or Eudragit L100 55 exhibited both the characteristic peaks of the drug and respective polymer, except Eudragit L100. The spectra of ASDs showed significant changes. For the ASD formulated by HPMCAS LG, the C=O stretching peak at 1711 cm^−1^ disappeared, and the C=O stretching (1652 cm^−1^ and 1611 cm^−1^) tended to broaden. The N–H stretching peak of nintedanib decreased significantly (Figure 9a). Moreover, the C=O stretching peak of HPMCAS LG at 1747 cm^−1^ shifted slightly to 1740 cm^−1^. The spectrum of HPMCAS MG–ASD was similar to that of HPMCAS LG–ASD, with a slight shift in the characteristic peak of the polymer from 1745 cm^−1^ to 1741 cm^−1^ (Figure 9b). These changes indicated the formation of H-bonds between the HPMCAS carbonyl group and the N–H bond in the nintedanib. In ASDs prepared with Eudragit L100 55 or Eudragit L100, the C=O stretching bands at 1711 cm^−1^ and 1652 cm^−1^ in nintedanib disappeared, along with the N–H stretching peak at 3414 cm^−1^. The C=O of Eudragit L100 55 (1735 cm^−1^) and Eudragit L100 (1725 cm^−1^) became weak, and the C=O stretching of Eudragit L100 55 at 1703 cm^−1^ blue-shifted to 1699 cm^−1^ (Figure 9c,d). These findings in the Eudragit–ASDs’ FT–IR spectra also suggested the H-bonding interactions between the N-H bond of nintedanib and the carbonyl group of Eudragit L100 55 or Eudragit L100. In addition, the peak at 1611 cm^−1^, as assigned to the stretching of C=O in the nintedanib band, weakened in Eudragit L100–ASD compared to Eudragit L100 55–ASD, implying that Eudragit L100 might provide stronger interactions with the drug. The contribution of hydrogen bonding between the drug and the enteric polymers might facilitate the inhibition of drug crystallization and precipitation [34,38].

### 3.6. In Vitro Dissolution with pH Shift

**Figure 10:**
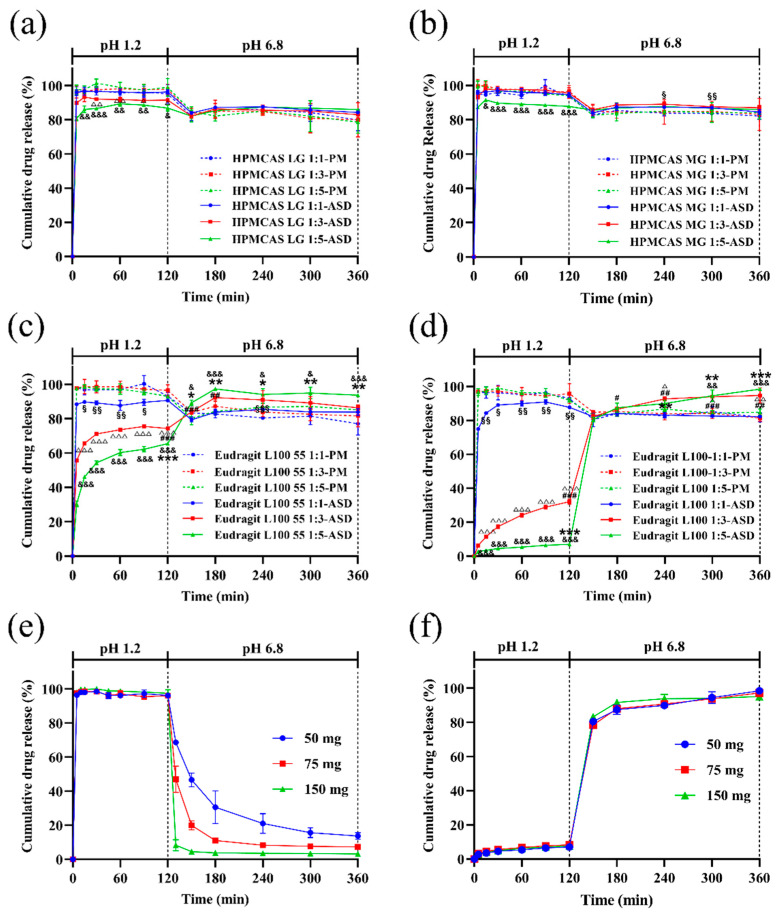
pH shift dissolution profiles in pH 1.2 for 120 min and pH 6.8 for 240 min (Mean ± S.D., *n* = 3). (**a**) HPMCAS LG–ASDs and PMs, (**b**) HPMCAS MG–ASDs and PMs, (**c**) Eudragit L100 55–ASDs and PMs, (**d**) Eudragit L100–ASDs and PMs, (**e**) nintedanib, (**f**) Eudragit L100–ASDs. 1:5 ASD group significantly different from the 1:1 ASD group: * *p* < 0.05, ** *p* < 0.01, *** *p* < 0.001; 1:3 ASD group significantly different from the 1:1 ASD group: ^#^ *p* < 0.05, ^##^ *p* < 0.01, ^###^ *p* < 0.001; 1:5 ASD group significantly different from the 1:5 PM group: ^&^ *p* < 0.05, ^&&^ *p* < 0.01, ^&&&^ *p* < 0.001; 1:3 ASD group significantly different from the 1:3 PM group: ^△^ *p* < 0.05, ^△△^ *p* < 0.01, ^△△△^ *p* < 0.001; 1:1 ASD group significantly different from the 1:1 PM group: ^§^ *p* < 0.05, ^§§^ *p* < 0.01, ^§§§^ *p* < 0.001.

Dissolution behaviors of ASDs with different ratios of nintedanib (50 mg) and polymers were examined in SGF for 120 min and then in SIF for 240 min. As depicted in Figure 10a–d, the type and content of enteric polymer largely influenced the drug dissolution rate. For HPMCAS (both LG and MG), more than 80% and 86% of nintedanib in ASDs was dissolved in SGF at 5 min and 120 min, while more than 94% of nintedanib was dissolved for all physical mixtures. Although there was no obvious retardation with HPMCAS LG and MG in drug release in SGF, the drug release of HPMCAS LG 1:3, 1:5, and MG 1:5 ASDs presented significantly lower values than that of the corresponding ratios of physical mixtures (*p* < 0.05, *p* < 0.01, or *p* < 0.001). A similar observation was made by Liu et al. [39]. When the pH of the medium changed to 6.8, the dissolved concentration of the drug slightly decreased, suggesting that the released drug precipitated (Figure 10a,b). Although the precipitates continued to redissolve, the drug concentration at 240 min in SIF still was lower than in SGF. In addition, HPMCAS LG–ASDs presented similar dissolution profiles to HPMCAS MG–ASDs at the ratios of nintedanib and polymers of 1:1 and 1:5 (*w*/*w*). Interestingly, HPMCAS ASDs exhibited a superior trend to maintain the supersaturation of nintedanib in pH 6.8 PBS but without marked significance, except for HPMCAS MG–ASDs at the ratio of 1:1 (Figure 10b). These results demonstrated that HPMCAS polymers inhibited the precipitation of weakly alkaline nintedanib due to the interaction of the drug and the polymers instead of the formation of ASDs, which coincides with a previous report [36].

The drug release profiles of Eudragit–PMs were similar to those of HPMCAS–PMs (Figure 10c,d). Compared to the initial concentration in SGF, the drug concentration tended to decrease with time in SIF. The release profiles of Eudragit–ASDs were different from the physical mixtures. The acid resistance of Eudragit–ASDs significantly increased in comparison with the corresponding ratio of Eudragit–PMs (*p* < 0.05, *p* < 0.01, or *p* < 0.001). The drug release from the Eudragit–ASDs in SGF also markedly declined with the ratio of polymer (*p* < 0.001). As illustrated in Figure 10c, the drug dissolution percentages in the Eudragit L100 55–ASD (1:3, *w*/*w*) group were significantly higher than those of the Eudragit L100 55–ASD (1:1) group at 150 min and 180 min in SIF (*p* < 0.01 or *p* < 0.001, Figure 10c). As for the 1:5 group, approximately 65% of the drug dissolved at 120 min, indicating that the larger ratio of Eudragit L100 55 further enhanced the anti–acid effect of the formulated ASD. Moreover, nintedanib in Eudragit L100 55–ASD (1:5) could dissolve and maintain a higher level of drug concentration at all time points compared to that of ASD (1:1) (*p* < 0.05 or *p* < 0.01). Compared to the PM groups, ASDs with the highest ratio of polymer (1:5) exhibited superior maintenance of nintedanib supersaturation in pH 6.8 PBS (*p* < 0.05 or *p* < 0.001). As for the ASDs with ratios of 1:1 and 1:3, an enhanced trend in the inhibition of drug precipitation could be observed, while only the group (1:1) at 240 min showed significance (*p* < 0.001). Compared to the related ratio of the Eudragit L100–PM group, the drug release levels in SIF were significantly improved at 240 min, 300 min, and 360 min (*p* < 0.05 or *p* < 0.01) for the 1:3 ASD group and at 300 min and 360 min (*p* < 0.01 or *p* < 0.001) for the 1:5 ASD group. Circa 32% of nintedanib in Eudragit L100–ASD (1:3) dissolved at 120 min, which significantly retarded drug release in comparison to the 1:1 group (*p* < 0.001), and the drug release percentages in pH 6.8 PBS buffer were significantly higher than those in the 1:1 group (*p* < 0.05, *p* < 0.01, or *p* < 0.001). The Eudragit L100–ASD (1:5) released approximately 7% of nintedanib at 120 min in SGF and 80% after 30 min and switched to SIF, indicating that Eudragit L100 demonstrated excellent stomach protection. The difference in dissolution rates in the Eudragit L100 55–ASD and L100–ASD in SGF might be attributed to the greater ionization rate of L100 55 due to its relatively low hydrophobicity [35].

Dissolution profiles for different doses of nintedanib are also shown in Figure 10e. Nintedanib completely dissolved in SGF within 5 min. After adjustment to SIF, rapid precipitation in the crystalline form generated a turbid suspension. The precipitation rate depended on the drug dose. The remaining percentage at 360 min was 14%, 7%, and 3% at 50, 75, and 150 mg, respectively. The cumulative dissolution decreased as the dose increased, which was in accordance with previous studies [10,40]. The nucleation rate strongly depended on the degree of supersaturation. A higher degree of supersaturation meant faster crystallization and precipitation of drug particles. In this regard, all four ASDs maintained the supersaturation state in SIF for 4 h, suggesting that these enteric polymers could effectively inhibit the precipitation of weakly basic drug nintedanib. As shown in Figure 10f, nintedanib dissolution profiles from the Eudragit L100–ASDs were similar among all three dosages, indicating that the formulated ASD system could maintain the supersaturation state in SIF. These results further demonstrated that Eudragit L100 dramatically provided both acid resistance and inhibition of precipitation for weak bases.

### 3.7. Stability under High Temperatures and High Humidity

**Figure 11:**
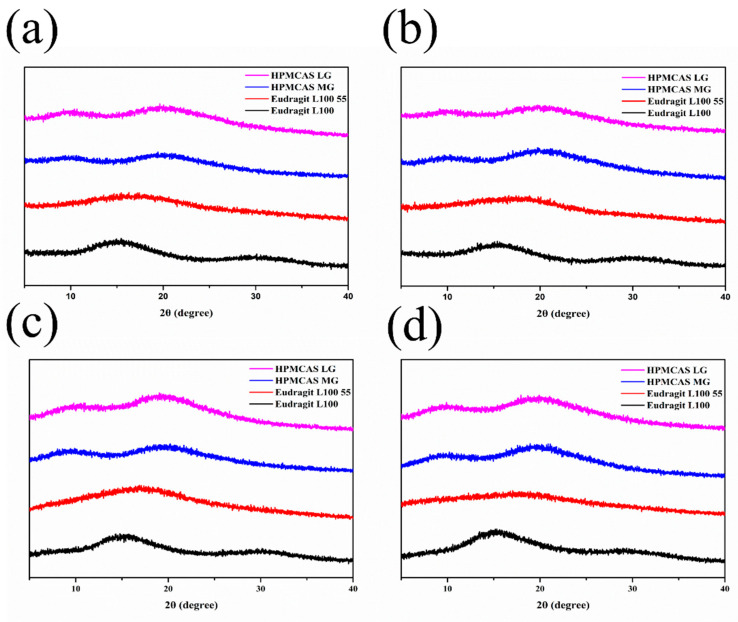
PXRD of ASDs at 92.5% ± 5% humidity and 60 °C ± 1 °C temperature. (**a**) 5 days (humidity), (**b**) 10 days (humidity), (**c**) 5 days (temperature), (**d**) 10 days (temperature).

**Figure 12:**
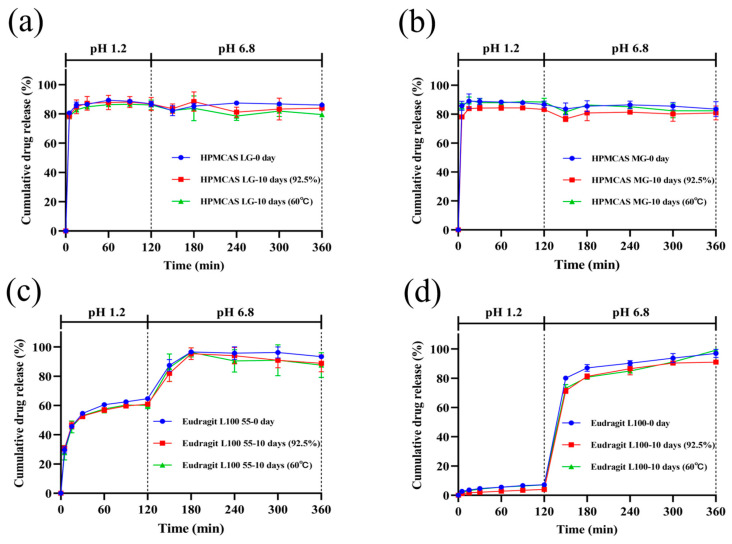
pH shift dissolution behavior of ASDs before and after being subjected to high temperature and high humidity for 10 days (mean ± SD, *n* = 3). (**a**) HPMCAS LG, (**b**) HPMCAS MG, (**c**) Eudragit L100 55, (**d**) Eudragit L100.

The physical stability of solid dispersions was evaluated by a stress test under 92.5% ± 5% humidity and 60 °C ± 1 °C temperature. As presented in Figure 11, the PXRD diffraction diagrams of the ASDs after the stability test were similar to the initial systems (Figure 7). No diffraction peak of nintedanib was detected, confirming that the drug in all four ASDs still existed in an amorphous state. Comparing the dissolution profile at 10 days with that at 0 days (Figure 12), the values of the *f*_2_ similarity factors were between 64 and 87, demonstrating the dissolution profile similarities (*f*_2_ > 50) among all the formulations. These results showed that using HPMCAS LG, HPMCAS MG, Eudragit L100 55, and Eudragit L100 as a matrix provided an amorphous form of nintedanib and it remained physically stable.

### 3.8. In Vivo Pharmacokinetics

**Figure 13:**
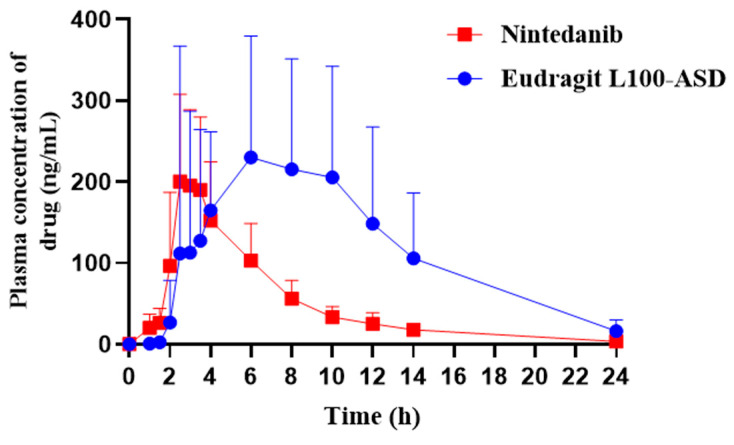
Mean plasma concentration–time curves of nintedanib after the oral administration of nintedanib and Eudragit L100–ASD in rats at a dose of 50 mg/kg (mean ± SD, *n* = 6).

The Eudragit L100–ASD was further chosen to investigate the effect of enteric polymers on the absorption of the weakly alkaline nintedanib. The plasma concentration–time profiles and main pharmacokinetic parameters are shown in Figure 13 and Table 2, respectively. For the Eudragit L100–ASD group, the plasma concentration was not detected until 2 h. Compared to the nintedanib group (2.8 ± 0.4 h), the peak plasma concentration was achieved at 5.3 ± 2.7 h. The *T*_max_ markedly increased (*p* < 0.05), indicating that Eudragit L100–ASD could resist gastric acid and delay the drug release in the GIT. The mean *C*_max_ for Eudragit L100–ASD (370.0 ± 159.6 ng/mL) was 1.5–fold higher than that of nintedanib (248.3 ± 70.4 ng/mL), but the lack of marked difference (*p* > 0.05) might be due to the high individual variation among rats. The AUC_0–24h_ for Eudragit L100–ASD (2710.6 ± 1479.4 ng/mL·h) was significantly greater than that obtained with nintedanib (1107.6 ± 292.3 ng/mL·h) (*p* < 0.05), indicating that the ASD formulation significantly improved the oral bioavailability (*F*_rel_ = 245%). No significant differences were observed for *t*_1/2_ values of Eudragit L100–ASD and nintedanib. However, the MRT of Eudragit L100–ASD significantly increased compared to the crystalline drug (*p* < 0.01), indicating that the enteric polymer extended the absorption period and provided a sustained plasma profile.

The reasons for the bioavailability enhancement by Eudragit L100–ASD are listed as follows: (1) the amorphous conversion of crystalline nintedanib improved the solubility of the weakly alkaline drug and facilitated dissolution in the intestinal fluid [12]; (2) the polymer Eudragit L100 inhibited the precipitation and recrystallization of the dissolved drug, and effectively maintained the state of drug supersaturation; (3) the maintenance of the supersaturation state could provide high energy, which maintained a high concentration gradient state to drive the drug to be transported across the intestinal membrane and promoted oral drug absorption [41]. Although the improved bioavailability was comparable to that of reported novel formulations [8,9], the excellent maintenance of supersaturation in the neutral medium in vitro only contributed to a limited augmentation, suggesting that other issues should be considered for further improvement [6,7]. Large inter–individual variation in the plasma concentration of nintedanib was also observed for the Eudragit L100–ASD group. As the amorphous drug was distributed throughout the Eudragit L100 matrix, the dissolution rate of the polymer in the gastrointestinal fluid controlled the drug dissolution. The intrinsic dissolution characteristics of Eudragit L100 and the relatively low volume of intestinal fluids might contribute to preventing the formation of high drug concentrations in the lumen, as demonstrated in the delayed *T*_max_ and prolonged MRT (Table 2). Individual differences, such as the pH value and volume of intestinal fluid, as well as the metabolism enzymes and transporters, influenced the drug absorption of Eudragit L100–ASD in vivo [42].

## 4. Conclusions

Poorly water–soluble, weakly basic drugs precipitate easily in the intestinal tract after the drug solution empties from the stomach, leading to low bioavailability. In the present study, pH–dependent soluble polymers, HPMCAS LG, HPMCAS MG, Eudragit L100 55, and Eudragit L100, have been compared for the solubility enhancement of nintedanib and supersaturation maintenance. All polymers were observed to increase the solubility and reduce precipitation rates relative to the growth rate of the drug alone in pH 6.8 PBS buffer. Compared to HPMCAS LG and HPMCAS MG, Eudragit L100 55 and Eudragit L100 were more effective. The stabilizing effects on supersaturation in a neutral medium may be related to the interaction of drug–polymer, which was confirmed by FT–IR. The dissolution results of four polymer–ASDs demonstrated that all polymers could effectively maintain the drug supersaturation, even at the lowest ratio. The pharmacokinetic characteristics of Eudragit L100–ASD further demonstrated that enteric polymer–based ASDs are an effective strategy to increase the oral bioavailability of nintedanib by improving the dissolution properties in the small intestine.

## Figures and Tables

**Table 1:** MRM condition for nintedanib and IS.

Compound	Precursor Ion > Product Ion (*m*/*z*)	DP (V)	EP (V)	CE (eV)	CXP (V)
Nintedanib	540.3 > 113.0	126	10	39	8
Carbamazepine (IS)	236.9 > 193.9	161	10	31	16

**Table 2:** The main pharmacokinetic parameters of nintedanib after oral administration of nintedanib and Eudragit L100–ASD in rats at a dose of 50 mg/kg (*n* = 6).

Parameters	Nintedanib	Eudragit L100–ASD
*T*_max_ (h)	2.8 ± 0.4	5.3 ± 2.7 *
*C*_max_ (ng/mL)	248.3 ± 70.4	370.0 ± 159.6
AUC_0–24h_ (ng/mL·h)	1107.6 ± 292.3	2710.6 ± 1479.4 *
*t*_1/2_ (h)	3.2 ± 0.8	3.6 ± 1.1
MRT (h)	6.3 ± 0.8	9.3 ± 1.4 **
*F*_rel_ (%)	/	245%

* *p* < 0.05, ** *p* < 0.01: significantly different from the nintedanib group.

## Data Availability

All data are included in the manuscript.
